# Specificity and Plasticity of the Functional Ionome of *Brassica napus* and *Triticum aestivum* Exposed to Micronutrient or Beneficial Nutrient Deprivation and Predictive Sensitivity of the Ionomic Signatures

**DOI:** 10.3389/fpls.2021.641678

**Published:** 2021-02-10

**Authors:** Aurélien D’Oria, Galatéa Courbet, Aurélia Lornac, Sylvain Pluchon, Mustapha Arkoun, Anne Maillard, Philippe Etienne, Sylvain Diquélou, Alain Ourry

**Affiliations:** ^1^UMR 950 Ecophysiologie Végétale, Agronomie et Nutritions N, C, S, Normandie Université, UNICAEN, INRAE, Caen, France; ^2^Laboratoire de Nutrition Végétale, Centre Mondial de l’Innovation, Le Groupe Roullier, Saint-Malo, France

**Keywords:** ionome, ionomic signatures, nutrient deficiency, nutrient interactions, rapeseed, wheat

## Abstract

The specific variation in the functional ionome was studied in *Brassica napus* and *Triticum aestivum* plants subjected to micronutrient or beneficial mineral nutrient deprivation. Effects of these deprivations were compared to those of macronutrient deprivation. In order to identify early events, plants were harvested after 22 days, i.e., before any significant reduction in growth relative to control plants. Root uptake, tissue concentrations and relative root nutrient contents were analyzed revealing numerous interactions with respect to the 20 elements quantified. The assessment of the functional ionome under individual mineral nutrient deficiency allows the identification of a large number of interactions between elements, although it is not totally exhaustive, and gives access to specific ionomic signatures that discriminate among deficiencies in N, P, S, K, Ca, Mn, Fe, Zn, Na, Si, and Se in both species, plus Mg, Cl, Cu, and Mo in wheat. Ionome modifications and components of ionomic signatures are discussed in relation to well-known mechanisms that may explain crosstalks between mineral nutrients, such as between Na and K, V, Se, Mo and S or Fe, Zn and Cu. More surprisingly, when deprived of beneficial nutrients such as Na, Si, Co, or Se, the plant ionome was strongly modified while these beneficial nutrients contributed greatly to the leaf ionomic signature of most mineral deficiencies.

## Highlights

Tightly regulated, the functional ionome is tissue and species specific, but shows a high degree of plasticity when plants are exposed to micronutrient and beneficial nutrient deprivation, resulting from numerous crosstalks between nutrients. Some highly sensitive specific signatures for each deprivation are outlined in this study.

## Introduction

The whole elemental composition of plants, also defined as the ionome, is species, genotype and tissue specific (Shakoor et al., 2016; Neugebauer et al., 2018), and while it is supposed to be tightly regulated, it can also be modified under changing climatic conditions (Soares et al., 2019) or when plants are facing numerous abiotic stresses such as atmospheric CO_2_ enrichment (Loladze, 2014), mineral deficiencies or drought (Etienne et al., 2018). The way that macronutrient deficiencies modify the plant ionome has been recently described in a companion paper (Courbet et al., 2021) using *Brassica napus* and *Triticum aestivum*. It highlighted numerous negative and positive interactions between macronutrient, micronutrient, and beneficial nutrients. Novel interactions were reported, for example, between S and vanadium (V), with a stimulation of vanadate uptake in plants cultivated under S deficiency that occurred alongside enhanced molybdate and selenate uptake, which had been described previously (Shinmachi et al., 2010; Maillard et al., 2016a; Courbet et al., 2019). Furthermore, the companion paper also highlighted a negative interaction between N and Na that was also strengthened using data extracted from the Ionomic Hub (Salt et al., 2008) with knockout Arabidopsis lines for genes encoding nitrate transporters, suggesting that NO_3_^–^ uptake and Na^+^ influx were connected.

Numerous studies have already indicated negative effects of micronutrient deficiencies on physiological and agronomic parameters, such as strong decreases in plant growth and grain yield and quality (Allen, 2000; Fageria et al., 2008; Rawat et al., 2013). Micronutrient and beneficial nutrients are thought to be required for many metabolic functions [for a review see Andresen et al. (2018)]. For example, plants need metal elements like manganese (Mn), iron (Fe), nickel (Ni), copper (Cu), zinc (Zn), and molybdenum (Mo) to operate in energy metabolism (Dalcorso et al., 2014; Andresen et al., 2018), photosynthesis (van Oijen et al., 2004), the mitochondrial respiratory chain, gene expression regulation (Dalcorso et al., 2014), hormone synthesis, Mo cofactor (MoCo) synthesis (Maillard et al., 2016b), and protein structure. In order to maintain nutritional homeostasis in plants, a finely tuned balance of tissue mineral content is achieved through the regulation of root transporters (Andresen et al., 2018) coupled with a controlled translocation from roots to the shoot tissues partly through the Casparian strip (Barberon et al., 2016; Barberon, 2017). While relatively few studies have considered the effects of micronutrient or beneficial nutrient deficiency on the plant ionome, some interactions between micronutrients have been described from more focused experiments.

One of the most frequently described examples of micronutrient homeostasis concerns Fe and its interactions with other nutrients such as Ni, Cu, Zn, and cobalt (Co). In dicotyledonous plants, Fe concentration regulation is at least partly achieved by the fine tuning of Fe uptake from roots through the expression of iron regulated transporter 1 (IRT1), a member of the *Arabidopsis* ZIP (Zrt/Irt-like protein) metal transporter family (Henriques et al., 2002), which acts as a major high-affinity Fe transporter (Vert et al., 2002). This has been confirmed by the chlorotic appearance of the *irt1-1* knockout mutant of *Arabidopsis thaliana* (Barberon et al., 2014). However, IRT1 is not only involved in root Fe uptake, but also in the uptake of divalent cations like Ni, Cu, Zn, and Co, which occurs more specifically under iron limitation when IRT1 expression is up-regulated (Korshunova et al., 1999; Henriques et al., 2002; Nishida et al., 2012; Waters and Armbrust, 2013; Alejandro et al., 2020). Indeed, Nishida et al. (2012) have reported that IRT1 mediates Ni accumulation in *A. thaliana* hydroponic cultures under Fe deficiency. Similarly, Li et al. (2013) reported that ZmIRT1 gene expression was increased in maize cultivated under Fe and also under Zn deficiency. Only a few studies have shown Cu interactions with IRT1 transporter expression, such as Waters and Armbrust (2013), who found that *Arabidopsis* leaf Cu content increased under low Fe conditions together with increases in Ni and Zn leaf content. Finally, Co, which is assumed to be a beneficial element, but whose function in non-leguminous plants remains to be established (with no specific Co transporter described so far), is widely accumulated in plants grown on different soils (Jayakumar and Jaleel, 2009; Jayakumar et al., 2013). Only a small number of studies have suggested that the transport of Co might also be mediated by IRT1 (Korshunova et al., 1999; Zelazny and Vert, 2015). Thus, during the Fe deficiency response, Zn, Mn, Cd, Co, and Ni could be taken up at least by an over-expressed IRT1 transporter but this will require molecular evidences as other transporters are probably involved in these processes (Curie et al., 2000; Pinto and Ferreira, 2015; Sasaki et al., 2016; Alejandro et al., 2020). In excess, these elements required detoxification mechanisms, such as vacuolar transporter expression [reviewed by Ricachenevsky et al. (2018)] in *Arabidopsis* and rice (*Oryza sativa*). The up-regulation of IRT1 when plants face Fe deficiency highlights a complex network of regulation pathways that lead to mineral content fluctuations in plant tissues. This is further illustrated by the fact that about 50 known ionome genes (KIG) related to Fe accumulation were recently identify in *Arabidopsis* (Whitt et al., 2020).

Another example of networks involving several nutrients can be found with Mo. Once taken up, Mo is mostly used for MoCo synthesis (Bittner, 2014), which are involved in the structure of essential enzymes such as nitrate reductase, aldehyde oxidase, and xanthine dehydrogenase (Zimmer and Mendel, 1999). In mitochondria, biosynthesis of the MoCo (Kaufholdt et al., 2017) requires not only Cu (Billard et al., 2014), but also Fe (Bittner, 2014), Zn, and S (Maillard et al., 2016b). Consequently, Mo root uptake and its resultant tissue concentration is increased when plants experience S, Mn, Zn, Fe, or Cu deficiencies (Maillard et al., 2016b). Overall, the synthesis of MoCo can be considered as a crucial crosstalk where several interactions between macronutrients and micronutrients occur.

Other micronutrient root co-transporters have been reported in literature (Sasaki et al., 2016; Alejandro et al., 2020) as for example AtNRAMP1 (Curie et al., 2000) and OsNRAMP5 (Sasaki et al., 2012), which belong to the Natural resistance-associated macrophage protein (NRAMP) family. The latters were assumed to transport Mn as well as Cd and Fe, and are both up-regulated by −Fe treatment, as well as under −Mn condition for AtNRAMP1. In rice, root Si influx proteins, named Lsi1 (Ma and Yamaji, 2006; Ma et al., 2006) and members of the nodulin 26-like intrinsic protein III (NIP III) family have been reported to transport not only Si but also Se (Zhao et al., 2010). These examples illustrate the need to take into account a full functional ionomic analysis when considering plant adaptations to micronutrient and beneficial mineral nutrient deficiencies with the objective of identifying all potential interactions. We assumed that total deprivation of a micronutrient [boron (B), chlorine (Cl), Mn, Fe, Ni, Cu, Zn, or Mo], or a beneficial element [sodium (Na), silicon (Si), Co, or selenium (Se)] in rapeseed and wheat grown in hydroponic culture had the potential to reveal numerous interactions within the two contrasting species. The first objective of this work was to identify these interactions and to determine the mechanisms involved. Therefore, the tissue ionomic composition was quantified before the appearance of any significant growth reduction as the approach to evaluating plant net uptake since deprivation, specific root accumulation (as a proxy of root to shoot translocation of nutrients) and tissue concentrations.

To study large ionomic datasets, multivariate analyses have been used to establish ionomic signatures of physiological responses to abiotic stress and plant growth stage (Baxter et al., 2008; Salt et al., 2008; Wu et al., 2013). For example, Baxter et al. (2008) showed that the ionomic signatures of *A. thaliana* could provide robust information to detect plant physiological responses to a specific environmental modification. Therefore, following partial least squares discriminant analysis (PLS-DA) of the ionomic data, the second objective of this work was, to establish tissue relevance in terms of sensitivity and specificity to characterize the specific ionomic signatures resulting from macronutrient (data extracted from companion paper Courbet et al., 2021), micronutrient or beneficial mineral nutrient deficiencies. Finally, these ionomic signatures were compared between the two species and their relevance tested using an independent dataset derived from Maillard et al. (2016a).

## Materials and Methods

### Plant Materials and Growth Conditions

*Brassica napus* (cv. Trezzor) and *T. aestivum* (cv. Bagou) were grown in a greenhouse (20°C day/15°C night) at Caen Normandy University (France) between February–March for rapeseed and April–May for wheat. Seeds of both species were germinated on perlite over demineralized water for 4–5 days in the dark followed by 2 days under natural light. After emergence of the first true leaf, seedlings were transferred to hydroponic conditions in a complete nutrient solution and were then split into 10 plants groups, each group grown in a 10 L plastic container. The plants were exposed to natural light and supplemented with high-pressure sodium lamps (HPS 400 Watt, Hortilux Schréder, Monster, Netherlands) at 350 μmol m^–2^ s^–1^ over a 16 h day/8 h night photoperiod. The composition of the nutrient solution used above was derived from Maillard et al. (2015) and adapted according to Courbet et al. (2021) to obtain a plant ionomic composition as close as possible to the ionome obtained with field grown plants, and comprised the following: 1 mM KNO_3_, 1.25 mM Ca(NO_3_)_2_, 0.2 mM KH_2_PO_4_, 0.4 mM MgSO_4_, 0.5 μM NaFe-EDTA, 50 μM NaFe-EDDHA, 10 μM H_3_BO_3_, 3 μM MnSO_4_, 3 μM ZnSO_4_, 0.7 μM CuSO_4_, 0.008 μM (NH_4_)_6_Mo_7_O_24_, 0.1 μM CoCl_2_, 0.15 μM NiCl_2_, 0.9 mM Si(OH)_4_, 0.5 mM CaCl_2_, 0.1 mM KCl, 0.01 μM Na_2_Se0_4_, 0.1 mM K_2_SO_4_, and 0.2 mM Na_2_SiO_3_ buffered to pH 6.8 with 0.36 mM CaCO_3_. It must be pointed out that among the constitutive elements of the plant functional ionome, vanadium (V) and aluminum (Al) were not provided directly, assuming that traces present in the other compounds used in the nutrient solution were sufficient. In order to maintain optimal nutrition conditions, this solution was tested with NO_3_^–^ strips (Macherey-Nagel, Düren, Germany) every day during plant growth and was renewed when NO_3_^–^ depletion reached 30% of the initial concentration. Overall, nutrient solutions were changed every 4–5 days with younger plants and every 2 days when plant biomass and therefore growth were maximal.

After 18 and 24 days of growth with complete solution for wheat and rapeseed, respectively, individual deprivations of eight micronutrients (B, Cl, Mn, Fe, Ni, Cu, Zn, and Mo) or four beneficial elements (Na, Si, Co, and Se) were imposed. A harvest of control plants was performed just before the application of nutrient deficiency, which corresponded to day 0 (D_0_) of the deficiency experiments. In order to separate tissues that developed before or during deficiencies, marker pen was used to record the level of growth by marking the last-developed leaves and petioles in wheat and rapeseed, respectively, before starting the deprivation treatments. The compositions of nutrient solutions used for individual micronutrient or beneficial nutrient deprivations are provided in the Supplementary Data 1 and were optimized to reduce the effect, as much as possible, of accompanying cations and anions of the deprived nutrient. Two plastic containers (20 plants in total) were used for each individual deficiency or control experiment.

On the day of deficiency application (D_0_), control plants were harvested as five replicates of four plants each to ensure enough material for subsequent analysis. After 22 days of deficiency (D_22_), before any significant decrease in plant growth, plants were randomly harvested for each condition using a pool of two plants per replicate (five replicates per condition). The duration of deficiency was adapted from Maillard et al. (2016a) who reported that at least 30 days of micronutrient deprivation were required to cause a significant growth reduction of plants in these conditions Therefore, the effects of individual deficiencies on the uptake of other nutrients could be analyzed without the interacting effects of reduced plant growth.

Tissues were harvested as follows: for both species, roots were separated from leaves; for rapeseed, leaves and petioles were also spit. Tissues that were present before nutrient deficiency were separated from new tissues that emerged after D_0_ and thus had developed during nutrient deficiencies. For both species and throughout the manuscript, the tissues present before deficiency have been described as old leaf blades (OLBs) or old petioles (OPs), while those whose development occurred during deficiency have been designated young leaf blades (YLBs) and young petioles (YPs). The fresh weight of each sampled tissue was recorded and sub-sampled into two homogeneous batches, one was stored at −80°C for molecular analysis and the second was dried at 65°C (72 h) for dry weight determination and used for further elemental concentration (i.e., ionome) quantification.

### Elemental Content Analysis and Calculations

All the analytical methods used were previously detailed in Maillard et al. (2016a) and Courbet et al. (2021). Briefly, all dried sampled were ground to a fine powder using 0.4 mm diameter stainless steel beads in an oscillating grinder (Mixer Mill MM400, Retsch, Haan, Germany). Most of the macronutrient (Mg, P, S, K, and Ca), micronutrient (B, Mn, Fe, Ni, Cu, Zn, and Mo), and beneficial elements (Na, Co, V, and Se) were quantified, after previous acid digestion of dry weight samples (about 40 mg), with high-resolution inductively coupled plasma mass spectrometry (HR-ICP-MS, Element 2^TM^, Thermo Scientific) and using internal and external standards. For total N concentration, 1.5 mg of fine powder were analyzed by using continuous flow isotope mass spectrometry (IRMS, Isoprime, GV Instruments, Manchester, United Kingdom) linked to a C/N/S analyzer (EA3000, Euro Vector, Milan, Italy). An x-ray-fluorescence spectrometer (XEPOS, Ametek, Berwyn, PA, United States) was used to quantify Cl, Si, and Al (Aluminum) from calibration curves from approximately 1 g of dry weight powder.

The quantity (Q) of each element in each harvested tissue (i) was calculated using the following equation:

(1) $$Q=E_{i_{D}}\times DW_{i_{D}}$$

where *E* is the elemental concentration (ppm) in a given tissue *i*, at each harvest day D (D_0_ or D_22_) and DW is the corresponding dry weight.

The net uptake (NU) of a given nutrient during one specific deficiency, i.e., between D_0_ and D_22_, can be estimated by the following equation:

(2) $$\mathrm{NU}=\sum_{i=1}^{n}Q_{i_{D22}}-\sum_{i=1}^{n}Q_{i_{D0}}$$

where *Q* is the amount of an element at day D of the plant tissue *i*, with n corresponding to three (roots, OLBs, and YLBs) or five (roots, OLBs, OPs, YLBs, and YPs) tissue types for wheat and rapeseed, respectively.

In this study, to assess the specific effect of a given deficiency, some of the results were expressed relative to control, using the ratios: $\frac{E_{deprived}}{E_{control}}$ or $\frac{NU_{deprived}}{NU_{control}}$

### RNA Extraction, Reverse Transcription (RT), and Quantitative PCR (q-PCR) Analysis

In order to evaluate the potential for modulation of *BnaIRT1* gene expression by different nutrient deficiencies, total RNA was extracted from 200 mg of fresh root material. Frozen samples were powdered with a pestle in a mortar containing liquid nitrogen. 750 μl of extraction buffer [0.1 M TRIS, 0.1 M LiCl, 0.01 M EDTA, 1% SDS (w/v), pH 8] and 750 μl of hot phenol (80°C, pH 4.3) were added to the root powder and vortexed for 40 s. After addition of 750 μl of chloroform/isoamyl alcohol (24/1; v/v), the mixture was centrifuged at 15,000 × *g* for 5 min at 4°C. The supernatant was recovered and transferred into 750 μl of 4 M LiCl solution (w/v) and stored at 4°C overnight. Then, samples were centrifuged at 15,000 × *g* for 20 min at 4°C. The supernatant was eliminated and the pellet was suspended in 100 μl of sterile water. Extracted RNA was purified with an RNeasy mini kit (Qiagen, Courtaboeuf, France). Quantification of total RNA was assessed with spectrophotometry at 260 nm (BioPhotometer, Eppendorf, Le Pecq, France).

A 1 μg of total RNA was converted to cDNA with an iScript cDNA synthesis kit (Bio-Rad, Marne-la-Coquette, France) for reverse transcription (RT). cDNA was diluted at 100×.

For quantitative PCRs (q-PCRs), 11 μL of Master Mix was prepared with 0.5 μM of primers, SYBR Green 2× (Bio-Rad, Marne-la-Coquette, France) and 2 μL ultrapure water before adding 4 μl of diluted cDNA and using a real-time thermocycler (CFX96 Real Time System, Bio-Rad, Marne-la-Coquette, France). The three incubation steps in the program were: (i) activation at 95°C for 3 min; (ii) 40 cycles of denaturation at 95°C for 10 s; (iii) and an extension step at 60°C for 40 s. For each pair of primers, the amplification specificity was monitored by the presence of a single peak in the melting curves within the thermocycler program and by sequencing the q-PCR product (Biofidal, Vaulx-en-Velin, France). After primer validation, the relative expression of the genes in nutrient deficiency samples was compared with the control sample and calculated with the delta Ct (ΔΔCt) method (Livak and Schmittgen, 2001): relative expression = 2^–ΔΔ*Ct*^ with Ct corresponding to the threshold cycle determined for each gene in the exponential phase of PCR amplification,

$$\Delta \Delta Ct=\Delta Ct_{sample}-\Delta Ct_{control}\text{and}$$

$$\Delta Ct=Ct_{targetgene}-Ct_{referencegene}.$$

For q-PCR amplification the following primers were selected: *EF1* (Forward: 50-GCCTGGTATGGTTGTGACCT-30; Reverse: 50-GAAGTTAGCAGCACCCTTGG-30) and *18sRNA* (Forward: 50-CGGATAACCGTAGTAATTCTAG-30; Reverse: 50-GTACTCATTCCAATTACCAGAC-30) as housekeeping genes. *BnaIRT1* (Forward: 50-GCGTCAAGATGCAGATCAAGTGTT-30; Reverse: 50-GTTTTGAGTTCCACAACGAAATCC-30) was the target gene.

### Statistical Analysis

Data were based on five independent replicates. Thus, elemental concentration (E; ppm) and quantity (Q; mg or μg) are indicated as the mean ± SE (standard error) for *n* = 5. For each nutrient, net uptake (NU) is given as the mean ± SE for *n* = 25, considering all random subtractive combinations of nutrient quantity between five replicates at D_22_ and D_0_, according to the previously indicated Eq. 2.

Statistical analyses were performed using R software (version 3.5.1: R Core Team, 2019) and RStudio (version 1.3.959: RStudio Team, 2020). Significant differences among all treatments were established using the non-parametric Kruskal–Wallis test and the significant difference of the means between control and a specific mineral deprivation were determined using the non-parametric Wilcoxon test. The Dunn’s *post hoc* test was used for multiple comparisons of groups, especially to compare the accuracy level of the four PLS-DA prediction methods cited below. Heatmaps were generated using the gplots package (version 3.0.1.1) with a color gradient representing values relative to control plants between 0.2 (blue = low) and 5 (orange = high). Blank cells in the heatmaps corresponded to non-significant variations relative to control plants.

Multivariate analysis methods offer the ability to reduce the dimension of large data, where the number of variables (i.e., genes, proteins, metabolites or the elements tested here) widely exceeds the number of samples, via new variables (components) defined as combinations of all the original variables. The purposes here were: (i) to study whether the ionome could be a relevant tool to reveal plant nutritional status and whether or not it possesses enough information for multiclass predictions; (ii) and to detect ionomic signatures for each deficiency, highlighting the weight of each element in these signatures. For this purpose, data from this study about micronutrient and beneficial element deprivations, as well as data from a companion paper (Courbet et al., 2021) dealing with macronutrient deprivations were used in the following steps.

**FIGURE 1:**
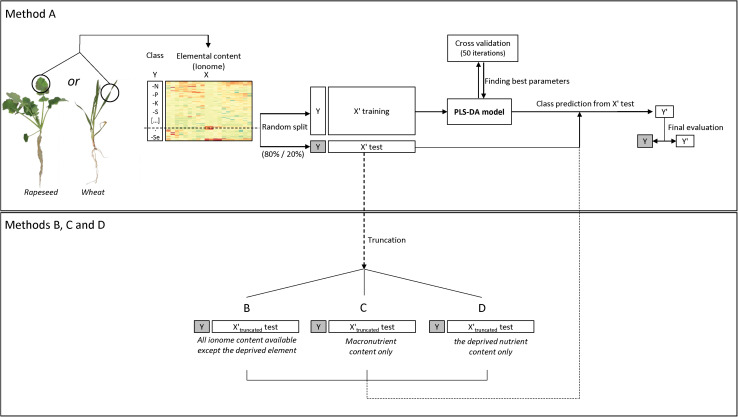
Flow diagram of the ionomic data analysis procedure using the PLS-DA model. The dataset containing the elemental concentrations (i.e., the ionome) (X) and deficiency classes (Y) was randomly split ten times into a training set and a test set (80/20 percent, respectively). Each time a five-fold and 50 repeat cross validation on the PLS-DA was used to measure model performance and find the best parameters (i.e., latent components to be retained) for class prediction. Thereafter a confusion matrix was used to assess prediction quality and the results were averaged after running the analysis ten times. Four methods and associated datasets were used (A) all ionomic data, (B) all ionomic data except the deprived nutrient, (C) macronutrient ionomic data, and (D) the deprived nutrient data.

After preliminary principal component analysis (PCA) to guide the modeling strategy, a PLS-DA model was used following the steps described in Figure 1 using the mixOmics R package (version 6.3.2) (Lê Cao et al., 2009; Rohart et al., 2017; Hervé et al., 2018) and the R caret package (version 6.0) (Kuhn, 2008). In this supervised classification method, the response variable Y is a class vector indicating the class of each sample (i.e., here the deprived element = deficiency). X is a matrix of predictors, composed here by the element raw concentration (ppm). The aim of the PLS-DA is to predict the class of new test samples based on a trained classification model (Rohart et al., 2017). Thus, the following process was repeated ten times.

The dataset (X) was randomly split into a training set (X′ training) and a test set (X′ test) (80/20 percentage, respectively) to limit overfitting and/or over-optimistic classification results (Brereton, 2006; Gromski et al., 2015), while keeping the original sample by class proportion in balance. This test set was used in the last prediction step by the PLS-DA model, using four different datasets: (i) using all data available (named thereafter method A) or, for exploratory purposes with a truncated test set of data (X′_*truncated*_ test) where (ii) the concentration of the deprived element was not considered (B), or (iii) only the concentration of macroelements were kept (C), or (iv) only the concentration of the deprived element was kept (D). The classification model was built using a five-fold and 50-repeat cross validation (CV) (Rohart et al., 2017) on centered and scaled data (because of the large differences in element concentration between macronutrient, micronutrient and beneficial nutrients). The optimal number of latent variables to retain was chosen based on the lowest classification error rate (CER) using the Mahalanobis distance calculation. The final PLS-DA model was used to predict classes (Y′) from the test set “X′ test” and to build a confusion matrix for comparison with real deficiency classes (Y) (Figure 1).

After the ten replication steps, averages were generated of the overall model prediction accuracy, the sensitivity (proportion of individuals predicted in a class that are correctly identified) and the specificity (individuals predicted as not belonging to a deficiency which does not actually belong to this deficiency) for each predicted class (deficiency). The variable importance in projection (VIP) of each predictor (i.e., element) contributing to determination of the predicted class (deficiency) was used to highlight specific ionomic signatures of the plant nutritional status. Hierarchical clustering on principal components (HCPC) with the Ward criterion was performed with the FactoMineR R package (version 1.41) to explore similarities between signatures.

Lastly, in order to validate the process and thus the results generated in this study, an external dataset was used for a supplemental validation. Consequently, the dataset generated by this experiment and the companion paper Courbet et al. (2021) was used following the same process, training a PLS-DA model to predict deficiency classes from the hydroponics experiments of Maillard et al. (2016a) where *B. napus* was grown under hydroponic conditions and included data for N, Mg, P, S, K, Ca, B, Mn, Fe, Cu, Zn, and Na.

## Results

### Root and Shoot Biomass

**FIGURE 2:**
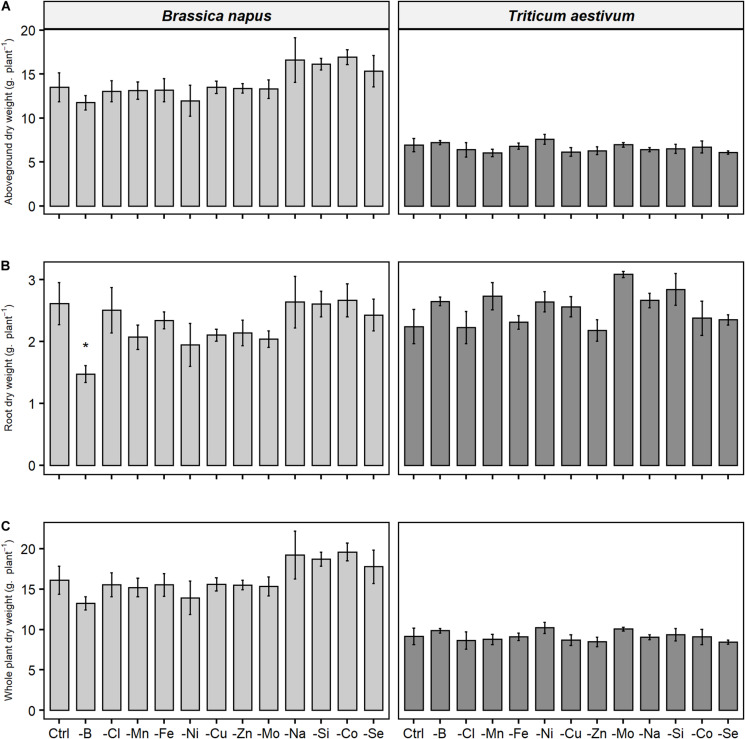
**(A)** Aboveground, **(B)** root, and **(C)** whole plant dry weight of *B. napus* and *T. aestivum* plants after 22 days of micronutrient or beneficial nutrient deprivation under hydroponic conditions. Data are given as the mean ± SE (*n* = 5) and significant differences between control and nutrient-deprived plants are indicated as follows: *, *p* < 0.05.

A 22-day deprivation of one of the micronutrients or beneficial elements had no effect on root or shoot biomass in either rapeseed or wheat (Figure 2), no visual symptoms causes by deficiency were recorded and photosynthetic activity was unchanged (unshown data) compared to the respective control plants. The only exception was found for rapeseed, which when subjected to B deprivation, demonstrated significant reduction in root dry weight, while the shoot biomass was only slightly lowered in comparison to control plants (not significantly). Micronutrient or beneficial element deprivations over the 22 days did not interfere with growth and thus growth was not considered as an explanatory variable.

### Relative Root Nutrient Content Under Micronutrient and Beneficial Nutrient Deprivation

**FIGURE 3:**
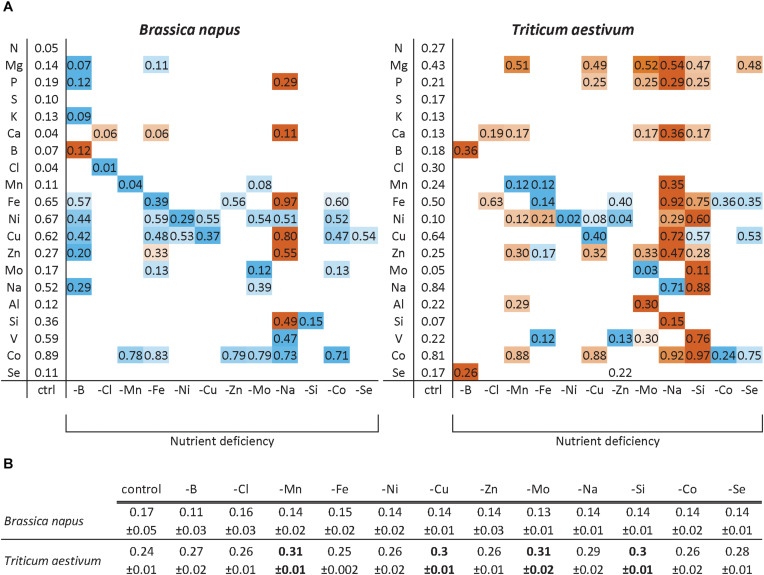
Relative root nutrient contents (RRNC) of *B. napus* and *T. aestivum* plants after 22 days of micronutrient or beneficial nutrient deprivation **(A)**. The RRNC was calculated as the ratio of nutrient content in roots/entire plant. A color gradient for large increases (red) or decreases (blue) in the RRNC are given only if they were significantly different from control plants for *p* < 0.01. The ratios of root biomass/whole plant biomass **(B)** are also given as the mean ± SE. for comparisons with RRNC, and only the values indicated in bold differ significantly from control plant biomass.

The relative root nutrient content (RRNC) corresponding to the ratio of roots to total plant content is given in Figure 3. First of all, the RRNC of N and S were unaffected by any of the nutrient deprivations in both species, which was also the case for K and Cl in wheat and Al and Se in rapeseed. Generally, the RRNC of the nutrient that was deprived was usually reduced compared to control plants, indicating that translocation from the roots to the shoots was favored. This was the case for nearly all nutrients except for B in both species. About 12 and 36% of B was kept in the roots under B deprivation versus 7 and 18% in control rapeseed and wheat, respectively. Moreover, under B deprivation, it should be noted that the root biomass was significantly reduced in rapeseed (Figure 2) leading to a lower root to whole plant biomass ratio (Figure 3B). In rapeseed, the RRNCs of individual nutrients were either unaffected or decreased by micronutrient or beneficial deprivation (Figure 3), with the exception of Na deprivation (increase of RRNC of P, Ca, Fe, Cu, Zn, and Si). This general trend can be illustrated for example under B (reduced RRNC of Mg, P, K, Fe, Ni, Cu, Zn, and Na) or Fe (reduced RRNC of Mg, Fe, Ni, Cu, Mo, and Co) deprivations. The trend was slightly different in wheat as there were greater increases in some RRNCs following deprivation of a specific micronutrient or beneficial nutrient. For example, under Si deprivation in wheat (Figure 3A), the relative root contents of Mg, P, Ca, Fe, Ni, Zn, Mo, Na, V, and Co were significantly higher than in control plants. In both species, under Na deprivation, the relative root contents of Mg, Ca, Mn, Fe, Cu, Zn, and Si were significantly higher than in control plants. Overall, the results show that the RRNC was not only a function of the root to whole biomass ratio as it can be significantly affected by the availability of some micronutrient or beneficial mineral nutrients.

### Relative Net Uptake of Mineral Nutrients and Their Concentration in Plant Tissues

**FIGURE 4:**
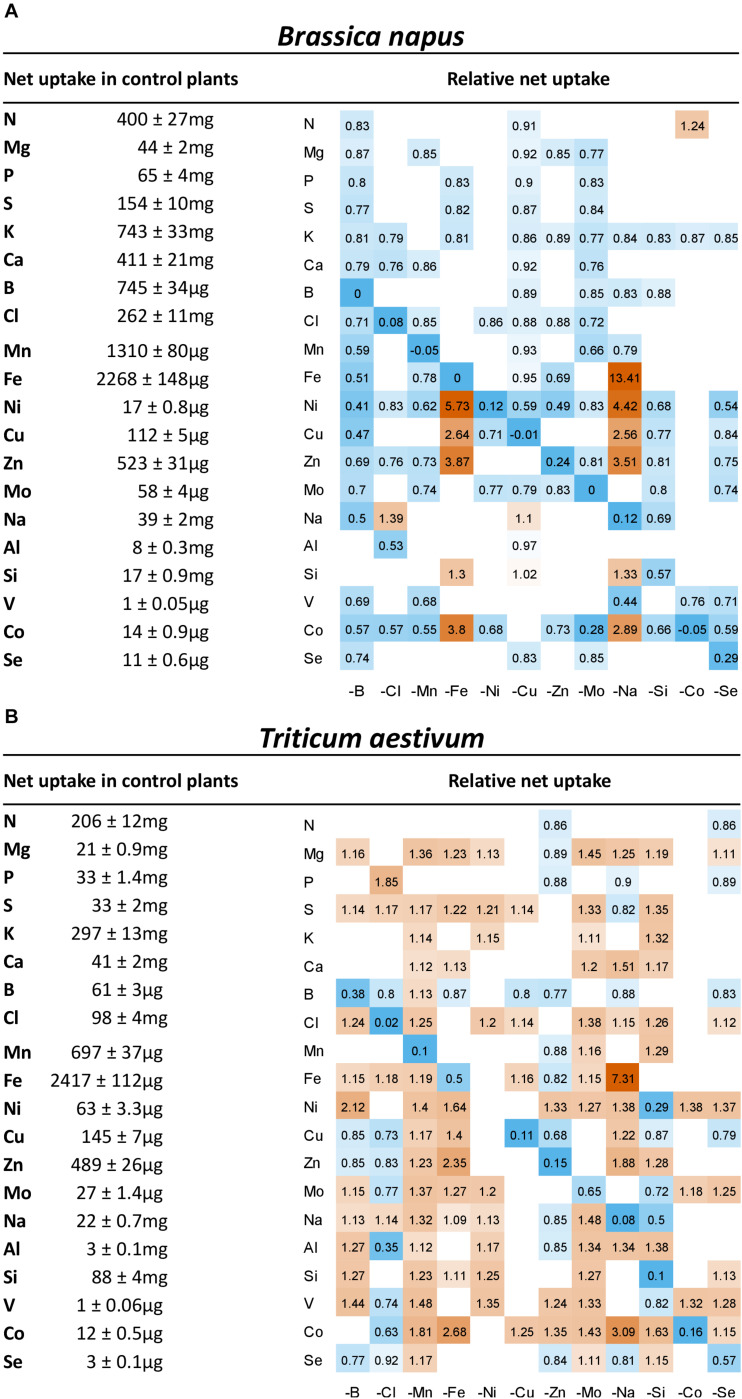
Heatmap of relative net uptake by **(A)** *B. napus* and **(B)** *T. aestivum* plants after 22 days of micronutrient or beneficial nutrient deprivation under hydroponic conditions. Relative net nutrient uptake was calculated (see section “Materials and Methods”) as the ratio of nutrients taken up by deprived plants/nutrients taken up by control plants. Only values significant for *p* < 0.05 are given, with the color gradient indicating values relative to control plants between 0.2 (blue = low) and 5 (orange = high). Blank cells in heatmaps corresponded to non-significant variations in net uptake compared to control plants.

The root uptake of nutrients, expressed relative to the uptake measured in control plants (Figure 4) was significantly affected under micronutrient or beneficial nutrient deprivation in both species, although with opposite trends in rapeseed (general decreases) and wheat (general increases). Overall, 103 negative and 14 positive interactions were found in rapeseed versus 46 and 99, respectively, in wheat.

Firstly, for most treatments, nutrients that were deprived had a relative net uptake close to 0, except for Si and Se for which some traces were probably found in the water used to make the nutrient solutions. For these elements, deficiency should be the consideration rather than deprivation. In wheat, the nickel-deprived treatment showed no significant decrease in net Ni uptake compared to control plants, so the Ni deprivation results must be considered with caution.

**FIGURE 5:**
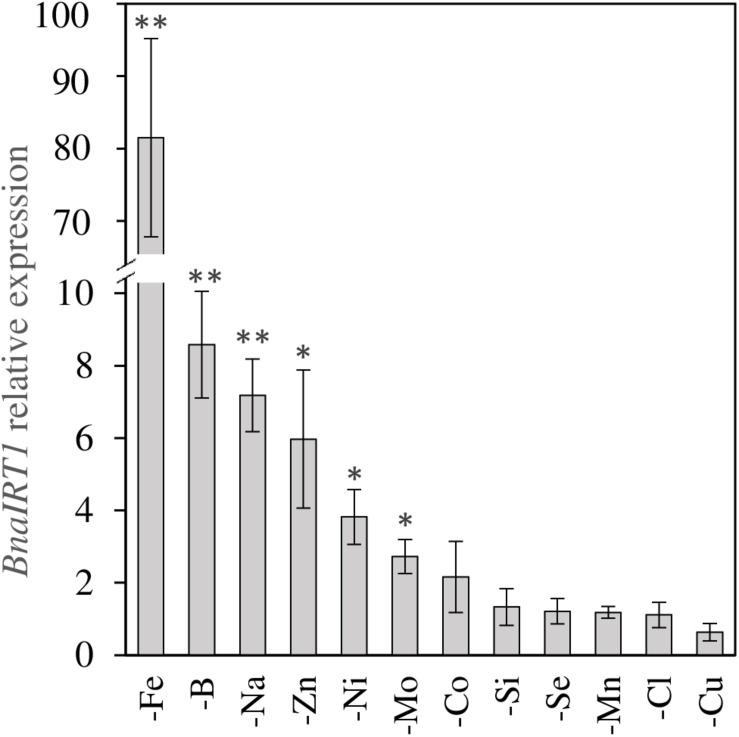
*BnaIRT1* gene expression relative to control plants in roots of *B. napus* subjected to micronutrient or beneficial nutrient deprivation for 22 days. Values are given as the mean ± SE (*n* = 5) and significant differences between control and nutrient-deprived plants are indicated as follows: ^∗^*p* < 0.05; ^∗∗^*p* < 0.01.

**FIGURE 6:**
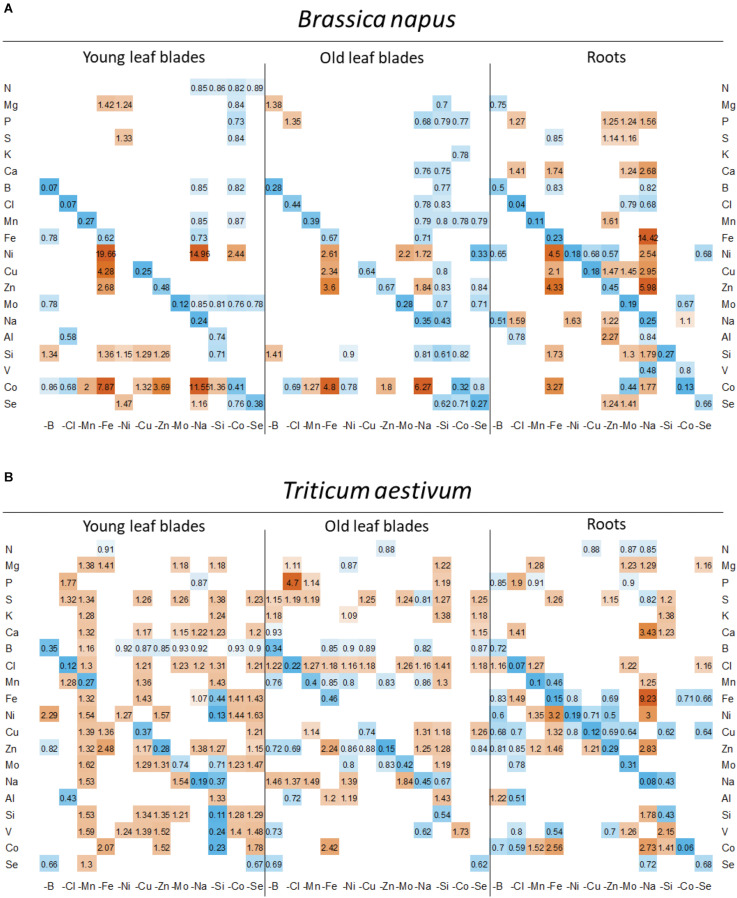
Heatmap of relative mineral nutrient concentrations in **(A)** *B. napus* and **(B)** *T. aestivum* plants after 22 days of micronutrient or beneficial nutrient deprivation under hydroponic conditions. Relative mineral nutrient concentration was calculated (see section “Materials and Methods”) as the ratio of the nutrient concentration in deprived plants/nutrient concentration in control plants. Tissues developed before or after the beginning of deprivation (D_0_) are indicated as “young leaf blades” (YLBs) and “old leaf blades” (OLBs), respectively. Only values significant for *p* < 0.05 are given, with the color gradient indicating values relative to control plants between 0.2 (blue = low) and 5 (orange = high).

Secondly, positive interactions affecting both species were observed, such as Fe deprivation, which increased the uptake of Ni, Cu, Zn, and Co. This was also the case under Na deprivation during which Fe uptake was also strongly increased (Figure 4). Since the assumption is that a root transporter encoded by the *BnaIRT1* gene takes up these nutrients, the expression of this gene has been quantified in rapeseed roots (Figure 5). The root expression of *BnaIRT1* was strongly and significantly increased under Fe, B, Na, Zn, Ni, and Mo deprivations, by 83, 8, 7, 6, 3.8, and 2.7 fold, respectively, relative to control plant roots. However, under Fe and Na deprivations only, this stronger expression led to an increase in root uptake of the above-mentioned nutrients in rapeseed roots (Figure 4A). Consequently, for Fe-deprived rapeseed plants, the concentrations of Ni, Cu, Zn, and Co were increased in roots and also in YLBs and OLBs (Figure 6A). The same trend was found under Na deprivation, but unlike the –Fe treatment, these elements were mainly accumulated in roots (Figures 3, 5). While the IRT1 expression in wheat roots was not quantified, it is possible that the same increase in gene expression occurred on the basis of the similar increased root uptake (Figure 4B) and tissue concentrations (Figure 6B) of Cu, Zn, and Co.

In wheat, a general trend for an increase in the uptake of most nutrients (see for example, Mg, S, Ca, or Cl uptake in Figure 4B) was found under most deprivation conditions and thus concentrations of macronutrients (except N), micronutrients and beneficial nutrients in roots, YLBs and OLBs (Figure 6B) increased, with the strongest trend in roots. Finally, the tissue concentrations (Figure 6) clearly showed that while Na deprivation strongly increased *BnaIRT1* expression in rapeseed (Figure 5), and the associated increased Fe uptake found in both species (Figure 4), the increased Fe concentration was only observed in the roots of rapeseed and wheat (Figure 5). This is perfectly in line with the higher Fe RRNC previously reported under Na deprivation (Figure 3), amounting to 97 and 92% in rapeseed and wheat, respectively, which was higher than the 65 and 50% recorded in controls of the two species, respectively. Similarly, a higher retention of Ca, Cu, Zn, and Si in roots obviously occurred under Na deprivation in both species (Figures 3, 4).

### Plasticity and Specificity of the Ionome Composition When Exposed to Mineral Nutrient Limitation

**FIGURE 7:**
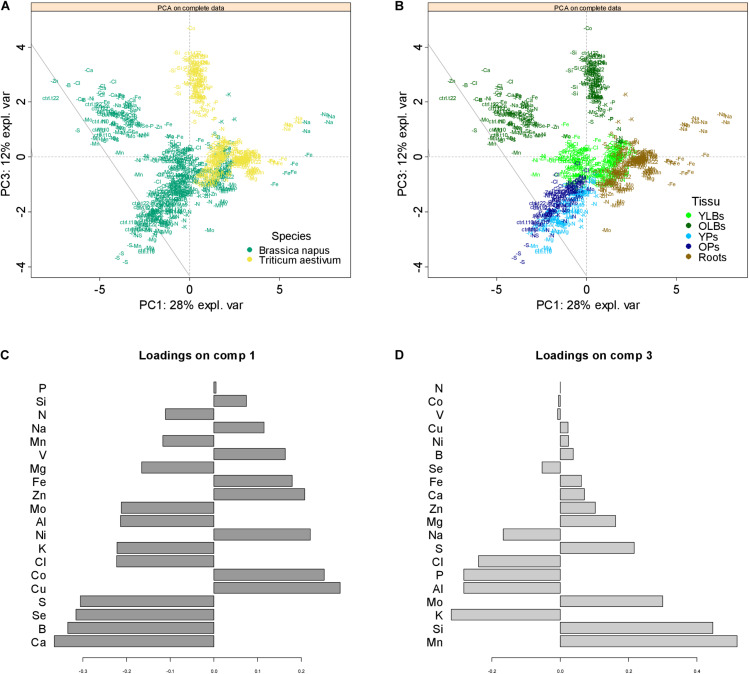
Principal component analysis (PCA) score plots of the complete elemental content data. Projection of the data onto the subspace spanned by components 1 (PC 1) and 3 (PC3), which are colored according to species **(A)** or tissue **(B)**, with each individual labeled with its treatment class (control or deprived plant). The contribution plots **(C,D)** depict the importance of each element in component 1 and 3, respectively, the bar length representing regression coefficients with either positive or negative signs. Variables are ranked by decreasing importance starting from the bottom.

The PCA of the entire elemental raw concentration is presented in Figure 7 with scatter plots of individuals colored by species (Figure 7A) or tissue (Figure 7B). On the first components, PC1 and PC3, which respectively explained 28 and 12% of the total variance, individuals of rapeseed and wheat (Figure 7A) as well as the tissues within each species (Figure 7B) were well discriminated (results on PC1 and PC2 are available in Supplementary Data 2). Loading vectors (i.e., the multivariate regression coefficient) indicating the importance of each element in component 1 (Figure 7C) suggested that the species-related discrimination was driven by Ca, B, Se, and S concentration in particular. On other hand, nutrients such as Mn, Si, K, and Mo, among others, contributed toward discriminating the different tissues analyzed (Figure 7D). This was supported by the elemental concentrations quantified in all tissues of both species (see Supplementary Data 3). These results led to an ionome analysis strategy that considered each tissue within each species independently.

The prediction accuracy is presented in Table 1 of the four methods of deficiency predictions that briefly considered a test set with all nutrients available for prediction (A), all nutrients available except the deprived nutrient (B), only macronutrients (C), or only the deprived nutrient (D). Overall, deficiency predictions by PLS-DA models for both species and all tissues were significantly more accurate in method A where all elements of the functional ionome were available (Table 1). For all predictions, the B method decreased the accuracy significantly compared to method A, indicating the deprived element weighting in the deficiency characterization. Further, all D method prediction results were significantly less accurate than the B method for wheat, and in most of the cases for rapeseed (except OPs and roots). Thus, the D method in its consideration of only the deprived element did not manage to reach the same level of prediction accuracy as the other elements (B) or the entire available ionome (A). Surprisingly for rapeseed, the prediction method based on the availability of all macronutrients (C) reached significantly lower accuracy compared to the process that considered only the deprived element (D). On other hand, results from methods C and D for wheat did not differ significantly. Consequently, the results were significantly more accurate with the addition of microelements (B) compared with the assessment using only the macronutrients (C).

**TABLE 1:** Overall accuracy percentage of the PLS-DA model predictions (after ten replications) (A) all ionomic data, (B) all ionomic data except the deprived nutrient, (C) the macronutrient ionomic data, and (D) the deprived nutrient data (see section “Materials and Methods” for details).

		Accuracy (%)			
Species	Tissue	A	B	C	D
*B. napus*	YLBs	94 a	68 b	29 c	58 b
	OLBs	92 a	60 a	30 b	**37** b
	YPs	95 a	**50** b	21 c	**45** b
	OPs	91 a	58 c	27 d	65 b
	Roots	82 a	**43** b	28 b	**47** b
*T. aestivum*	YLBs	94 a	61 b	29 c	33 c
	OLBs	**89** a	65 b	27 c	34 c
	Roots	96 a	64 b	32 c	31 c

*B. napus and T. aestivum tissues are indicated as follows: young leaf blades (YLBs), old leaf blades (OLBs), young petioles (YPs), old petioles (OPs), and roots. Percentages with the same letter for each tissue are not significantly different according to the Dunn’s test (p < 0.05; n = 10). Within each prediction method for each species, a bold value differs significantly from other tissue (from the highest accuracy according to the Dunn’s test; p < 0.05).*

For rapeseed in method A, the accuracy level did not differ significantly between all the tissues, even though the accuracy level obtained from root samples was lower. Young tissue grown during deficiency (YLBs and YPs) showed the highest level of accuracy. In contrast, in wheat the root samples allowed good prediction accuracy, whereas OLBs seemed to be the least relevant tissue for prediction with a significantly lower level of accuracy (Table 1).

Since the prediction results achieved from aboveground tissues were accurate for both species (Table 1), especially tissues that grew during deficiency, and also taking their harvest feasibility into consideration, YLBs seemed the most relevant for deficiency prediction. YLBs were therefore chosen to evaluate the predictions of PLS-DA models on micronutrient and beneficial element deprivation datasets from this study and macronutrient deprivations from the Courbet et al. (2021) companion paper. Finally, a supplemental validation was made that tested the PLS-DA prediction on an external validation dataset from Maillard et al. (2016a).

Thus, the sensitivity and specificity resulting from PLS-DA predictions for each class (deprived or control) are presented in Table 2 and the remaining tissues are available in the Supplementary Data 4. Overall, most of the deficiency classes were well classified and reached a sensitivity and specificity close or equal to one. It must be noted that Ni deficiency was the only exception for both species and reached only a 0.4 sensitivity, indicating that only 40% of nickel-deprived individuals were predicted as –Ni. The PLS-DA model was also good at predicting control plants from the D_22_ harvest and the harvest undertaken at D_10_ in the macronutrient deprivation experiments described in Courbet et al. (2021). The sensitivity and specificity was close to one for control treatments (except for D_22_ wheat where sensitivity only reached 0.8), which suggested that the elemental content allowed discrimination of control individuals from two time-separated harvests, and this indicated that ionome composition is also linked to the developmental stage.

**TABLE 2:** Sensitivity and specificity of PLS-DA prediction for each class predicted (control or deprived) in rapeseed and wheat young leaf blade (YLBs) samples after 10 days (D_10_) of macronutrient deprivation (Courbet et al., 2021) or 22 days (D_22_) of micronutrient or beneficial element deprivation.

	*B. napus*		*T. aestivum*		Maillard et al. (2016a)	
Class	Sensitivity	Specificity	Sensitivity	Specificity	Sensitivity	Specificity
control D_10_	0.9	1	1	1	NA	0.91
control D_22_	0.9	0.98	0.8	0.99	NA	0.84
−N	1	1	1	0.99	1	1
−Mg	1	1	1	1	1	1
−P	1	0.99	1	1	1	1
−S	1	1	1	1	1	1
−K	1	1	1	1	1	1
−Ca	1	1	1	1	0.50	1
−B	1	1	0.9	1	0.75	0.75
−Cl	1	0.98	0.9	1	NA	1
−Mn	1	0.99	1	1	1	1
−Fe	1	1	1	1	0	1
−Ni	0.4	1	0.4	0.99	NA	0.98
−Cu	0.9	1	1	1	0	0.93
−Zn	1	1	1	1	1	1
−Mo	1	1	0.9	0.99	0	0.95
−Na	1	1	1	1	NA	1
−Si	1	0.99	1	1	NA	1
−Co	0.7	0.99	0.9	0.99	NA	0.97
−Se	1	1	1	0.98	NA	0.84

*Sensitivity is defined as the ability of the test to correctly identify individuals in a class (i.e., deficiency), whereas specificity measure the test’s ability to predict that individuals do not actually belong to a deficiency. Briefly (see section “Materials and Methods”), a random subset of data was extracted (X′ test), the remaining data (X′ training) was used to train the PLS-DA, and the X′ test was used in the last step to compare classes predicted with it by the PLS-DA (Y′) from real deprivations applied to plants (Y). Results are presented as the mean (n = 10) from prediction method (A), considering the entire ionome. For supplemental validation purposes, the complete data from this study and a companion paper (Courbet et al., 2021) were used to train another PLS-DA model to try to predict the deficiency membership of rapeseed samples from hydroponic experiments conducted by Maillard et al. (2016a).*

Lastly, serving as supplemental validation, a PLS-DA model previously trained on YLBs sample data associated with the Courbet et al. (2021) companion paper, was used to predict the deficiency classes of the Maillard et al. (2016a) samples and led to accurate predictions for −N, −Mg, −P, −S, −K, −B, −Mn, and −Zn-treated plants (Table 2). A bias resulting from higher concentrations of Mo, Ca, and Fe in the nutrient solution used by Maillard et al. (2016a) led to incorrect classification of plants subjected to Mo, Ca, and Fe deprivations.

Remaining treatments involving Cl, Ni, and beneficial nutrient deficiencies were not available.

**FIGURE 8:**
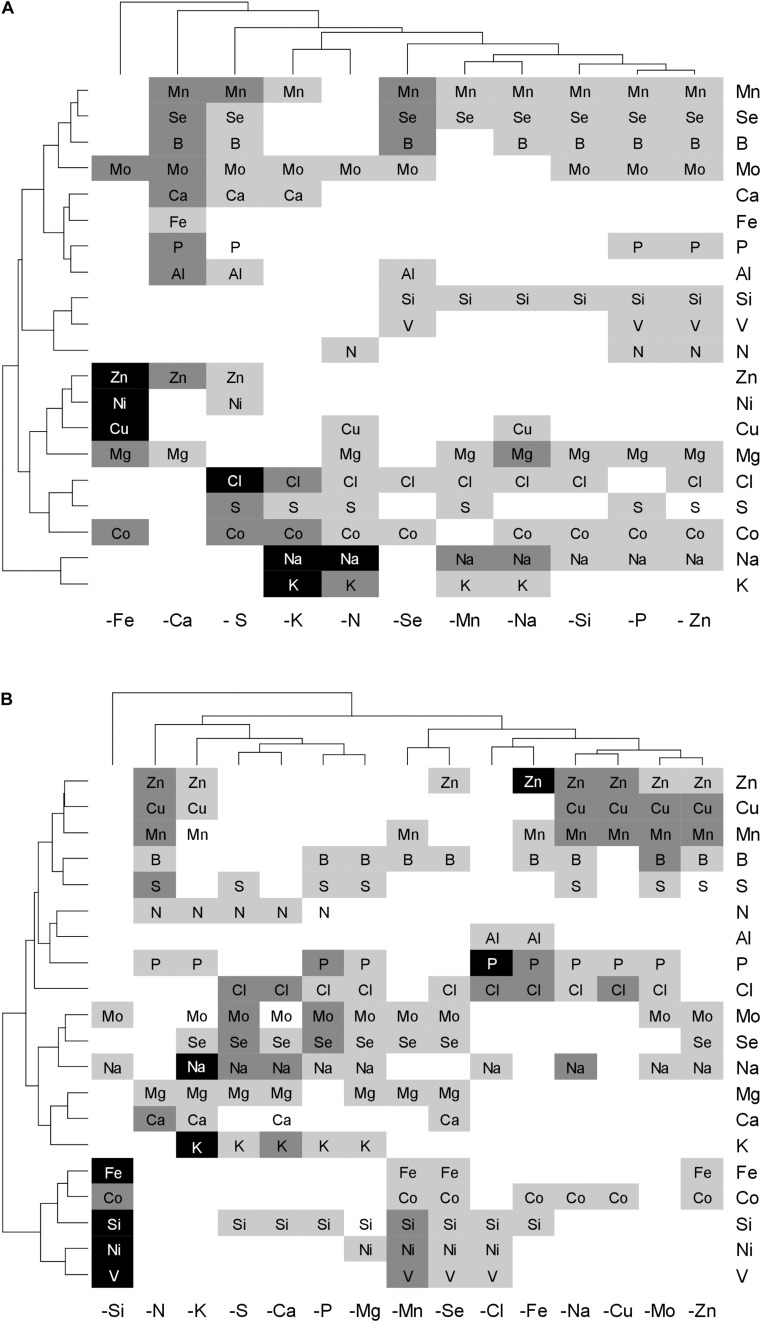
Heatmap of the elements with the highest VIP scores for each deficiency applied to *B. napus* **(A)** and *T. aestivum* **(B)** which were retrieved from the PLS-DA model on YLBs samples. Elements with VIP scores ≥1 are significant (≥1, light gray; ≥1.5, mid gray; ≥2, dark gray) and indicate the importance of each element (X variable) to predict deficiencies (Y). The specific combination of elements and their concentration variation (high or low in tissue) discriminate samples and generate signature.

In order to highlight the elements that contributed most to discriminating each deficiency in the PLS-DA on YLBs samples, grayscale heatmaps representing VIP are represented in Figure 8. Elements with a VIP greater than one were considered relevant for deficiency characterization when a clear pattern could be extracted from the PLS-DA. Overall, the obtained results were a good reflection of previous observations of YLBs content (Figure 6), indicating that concentration variations were specific enough to distinguished deficiencies from each other. As already described in Table 1, microelements seemed to be frequently retained as important elements and highly participate to establish a specific signature for each deficiency (Figure 8).

Hierarchical clustering on the VIP for wheat brought all of the macronutrient deprivation treatments closer. In contrast, for rapeseed the −Ca and −S patterns differed from the −K-N cluster and –P, indicating that for this species, macronutrient deprivations led to more specific ionome variations. As previously described for YLBs content, micronutrient deprivation in rapeseed caused fewer significant variations compared to wheat (Figure 6), limiting the identification of clear signature patterns in the PLS-DA components. Nevertheless, specific signatures for Mn, Fe, Zn, Na, Si, and Se deficiency for both species, plus Cl, Cu, and Mo for wheat were highlighted thanks to the PLS-DA approach. In addition, the rapeseed −Fe and wheat −Si treatments, whose global contents stand out from among the other treatments (Figure 6), showed strong signatures composed of a lower number of elements than the other deficiencies (Figure 8). On the other hand, although some signatures were composed of larger numbers of elements, some of them contained higher VIP scores, such as Na in K-deprived plants for both species (Figure 8). Within signatures, these elements thus contributed toward discriminating similar deficiencies.

In some cases, the deprived element was not found in the ionomic signature of its own deficiency, which was the case for −Fe in both species and −Zn in rapeseed (Figure 8). This could be explained by a low variation in the deprived element concentration in YLBs (Figure 6). Consequently, the ionomic signature seemed to be described as a qualitative ionomic variation, looking for elements contributing the most to discrimination, and associated with quantitative variations in concentrations of targeted elements. Finally, no specific ionomic signatures were found for the B, Co, and Ni deficiencies in either species or for Mg, Cl, and Mo deficiencies in rapeseed (Figure 8). Although they were well predicted (Table 2), these deficiencies showed too many similarities in their signatures with other deficiencies.

## Discussion

### The Ionome Discriminates Species, Tissues and Most Nutrient Deficiencies

The ionome appeared to be highly species and tissue dependent (Figure 7; Baxter et al., 2012), and the main variables contributing to their discrimination include Ca, B, Se, S, and Si (Figures 7C,D). As previously found in the Courbet et al. (2021) companion paper, rapeseed is characterized here by higher elemental concentrations, particularly B and S, than wheat, in line with previous studies showing that Poales were distinguished by their low Ca, B, and S concentrations relative to Brassicales (Broadley et al., 2004; Neugebauer et al., 2018). Hence, this species discrimination is related to an upper S concentration in Brassicaceae as previously described (Holmes, 1980; Zhao et al., 1993) while it has been shown that dicots require more Ca (Broadley et al., 2003) and B (Hu et al., 1996) for their cell wall composition. In the same way, cultivated Poaceae such as wheat, rice, barley, and maize are assumed to be Si accumulator species (Marschner, 2012) compared to Brassicales considered as low Si accumulators.

Furthermore, our results indicate that each tissue has a different ionome, demonstrating that each tissue should be considered independently within each species to avoid background noise while determining the signature.

We showed, in the purpose of an approach without *a priori*, that taking into account the plant tissue content of the deprived element only, did not allow discrimination of the 18 deficiencies examined in this study (Table 2) and in a companion paper (Courbet et al., 2021). In the same way, the sole use of the macronutrient content did not provide an as accurate prediction as a complete ionome assessment (Table 1). This was supported by the fact that among the macronutrients, N and P were considered to be poorly informative (Parent et al., 2013a, b). These results provide interesting evidence that combining micronutrients and beneficial elements in the analysis allows accurate identification of each applied deficiency (Table 2) and that analysis of the full functional ionome as a whole should be advocated for deficiency diagnosis (Cao et al., 2019). Considering each tissue independently, we also point out that leaves that have grown during the deficiency period (YLBs), and in particular the leaf blades, seem to be the most relevant tissue for the determination of nutritional status (Table 1). Indeed, as suggested by Baxter et al. (2008), the leaf ionome is the result of global genotype × environment interactions because it results from biochemical and physiological processes that originate from the roots to the shoots via apoplastic and symplastic transport, as well as vascular translocation and transpiration.

The functional ionome can then be used to design specific signatures of mineral deficiency resulting from physiological processes or to highlight crosstalks. Before any significant growth reduction (Figure 2), ionomic variation caused by a single deprived nutrient (Figures 3–5) can be considered as an early response. These ionomic variation gives access to a large source of interactions between elements possessing similar chemical properties or biological roles (Baxter, 2015), which in leaves were specific enough to provide deprivation signatures (Figure 8). Indeed, thanks to a PLS-DA approach, the whole macronutrient, micronutrient, and beneficial nutrient contents allows to correctly classified most of the deficiencies (Table 2) and to extract the most contributive elements which participate to characterized the specific signatures of N, P, S, K, Ca, Mn, Fe, Zn, Na, Si, and Se deficiency in both species, as well as Mg, Cl, Cu, and Mo in wheat (Figure 7). Si deprivation induces specific ionome variation in both species, nevertheless this signature being much more specific in wheat, which may be related to its Si accumulator behavior.

### Leaf Ionomic Signatures Are Determined by Specific Crosstalks, Some Known, Some Still Unknown

In rapeseed, micronutrient or beneficial element deficiency induces an overall decrease in the root uptake of mineral nutrients (Figure 4). With the exception of Na-deprived plants, this mainly resulted in the RRNC decreasing or remaining unchanged (Figure 3), while the YLBs content (Figure 6) showed only a few variations. The fact that only a few variations were measured suggests the existence of compensatory mechanisms, such as remobilization of endogenous reserves from source organs (e.g., mature leaves) to sustain growth of newly developed tissue (Malagoli et al., 2005; Abdallah et al., 2010), because numerous elements are assumed to be highly mobile (Etienne et al., 2018). The weak signal at the leaf level may explain why fewer signatures were found in micronutrient- or beneficial-nutrient-deprived rapeseed than in deprived wheat. Following micronutrient or beneficial nutrient deprivation in wheat, an overall increase in root uptake ultimately led to a global increase in elemental concentrations in YLBs. Hence, changes in the availability of one micronutrient or beneficial nutrient alone can greatly affect the uptake and concentrations of all other nutrients (Figures 6, 4) as well as their root/shoot partitioning (Figure 3).

Some deficiency signatures determined here can be explained by interactions that are well known in the literature. For example, because both Na^+^ and K^+^ are taken up by non-selective cation transporters such as HKT1-type (high affinity potassium transporter) or LCT1 (low-affinity cation transporter) (Schachtman and Liu, 1999), this crosstalk between both elements resulted in a large uptake of Na during K deprivation. Obviously Na, which accumulates in leaves (companion paper Courbet et al., 2021), is then one the most contributive element in −K signature (Figure 8).

Under S deficiency, Mo and Se are accumulated in rapeseed (Shinmachi et al., 2010; Maillard et al., 2016b), at least partly because of a strong induction of the expression of genes encoding root sulfate transporters (*Sultr1.1* and *Sultr1.2*). This may be explained by structural similarities among sulfate (SO_4_^2–^), molybdate (MoO_4_^2–^), and selenate (SeO_4_^2–^) that permit them to act as competitive anions for the sulfate transporters (Shinmachi et al., 2010; Schiavon et al., 2012). Hence, these elements were found in the specific signature determined for S deficiency in both species, as was accumulation of Cl. This increased Cl in −S treated rapeseed plants (Sorin et al., 2015) has been attributed to another crosstalk where Cl can partly osmotically replace SO_4_^2–^ during S-deficiency-induced remobilization from the vacuole. Additionally, Courbet et al. (2021) observed an increase in root V uptake in rapeseed and wheat subjected to S deficiency, suggesting that sulfate transporters might also take up vanadate (VO_4_^2–^). This was supported by quantification of V in Arabidopsis *sultr1.1* KO mutants and *sultr1.2 sel1-8* mutants. Nevertheless, because this accumulation mainly occurs in roots (Courbet et al., 2021) V therefore did not appear in the foliar −S signature (Figure 8). Indeed, the control of root to shoot translocation of elements such as V, which is probably due to the presence of the Casparian strip, may affect the leaf ionomic signature.

Furthermore, Fe, Cu, Mn, and Zn root uptake can be mediated by the iron regulation transporter, IRT1 (Grusak et al., 1999). Under Fe deficiency, an increase in IRT1 expression went hand-in-hand with an increased uptake and leaf accumulation of Mn, Co, Zn, and Cd, which together define a specific signature of the Fe nutritional status (Figure 8), as also reported by Baxter et al. (2008). This signature implies that studies focused on shoot Fe content alone might not provide evidence of an Fe deficiency because decreased Fe concentrations in growth media are not associated with fluctuations in shoot Fe content (Baxter et al., 2008), unlike the root Fe content. Consequently, Fe deficiency is an example where changes in content of the deprived element were mainly observed at the root level, whereas the associated leaf signature does not necessarily represent the element in question.

The overexpression of *BnaIRT1* gene observed in roots of *B. napus* plants cultivated under Fe, B, Na, Zn, Ni, and Mo deficiencies relative to the control (Figure 5) was concomitant with an increased root uptake of Ni, Cu, Zn, and Co (except when the element is deprived) in −Fe and −Na treated plants alone (Figure 4). Although IRT1 was not quantified in wheat and Fe acquisition is not governed by the same processes in dicotyledonous (Strategy I) and graminaceous monocotyledonous plants (Strategy II) (Marschner, 2012), the same trend was observed in both rapeseed and wheat. Na deprivation resulted in high RRNCs (Figure 3) and root concentration (Figure 6) of Fe, B, Na, Zn, Ni, and Mo but relatively few changes at the leaf level (except for Ni concentration). This suggested a relatively low level of translocation from roots to shoots under Na deficiency compared to Fe deficiency, and control of Cu, Zn, and Co transport via the Casparian strip.

The Casparian strip, which is a selectively impermeable structure made of suberin and lignin deposited by root endodermis cells (Naseer et al., 2012) can block passive flow of water and solutes in the root apoplast and consequently has a crucial role in selection of mineral nutrients in vascular plants (Barberon et al., 2016; Barberon, 2017). It has been shown that suberin accumulation in *A. thaliana* leads to water uptake disruptions coupled with a decrease in Ca, Mn, and Zn accumulation in the shoot (Baxter, 2009). In our results, the amounts of Fe, Cu, and Zn increased in roots but not in leaves in both species in association with an increase in IRT1 relative expression under Na deficiency. This suggested that transport is disrupted, which might be linked to a modification of the suberization of the Casparian barrier.

Finally, the overall results at the root uptake level, ionomic composition and derived signatures suggest multiple crosstalks between other mineral nutrients, but these are currently difficult to explain. The most striking examples concern beneficial mineral nutrients such as Na, Si, Co, and Se. Not only did their deprivation lead to massive changes in ionomic content in most tissues, and especially in wheat (Figure 6), they were also identified as elements that contributed to a large proportion of the leaf ionomic signatures (Figure 8). Their exact physiological roles in higher plants (particularly in the case of Co and Se) and their interactions with other mineral nutrients will require more focused research. Another example concerns the highly specific ionome signature in Si-deprived wheat for which Ni, Co, V, and Fe concentrations (Figure 8) are strongly decreased in young leaves (Figure 6). If the contrasted response of both species to Si deprivation could be explained by their opposite Si accumulator behaviors [high and low accumulator for wheat and rapeseed, respectively (Ma and Yamaji, 2006; Ma et al., 2006; Marschner, 2012)], the crosstalks between Si and above cited elements observed in wheat remain unknown and difficult to explain. A last example, as suggested in the companion paper (Courbet et al., 2021) the strong decrease in Na uptake in N- and P-deprived plants in rapeseed and wheat, which also appeared in their respective foliar signatures [supported by data provided by the Ionomic Hub (Salt et al., 2008)], highlights a link between N, P, and Na uptake. Indeed, there may be involvement of H^+^ ATPase during active transport of N and P whereby H^+^ efflux is coupled to root Na^+^ uptake (Courbet et al., 2021), but this remains to be characterized.

## Conclusion

In conclusion, this study demonstrated that deprivation of a single micronutrient or beneficial nutrient revealed numerous interactions between the mineral nutrients, leading to specific modifications of ionome composition as previously found with macronutrient deprivation. We showed that most mineral deficiencies could be diagnosed from the resulting leaf ionome. Based on the most contributive elements, the ionomic signatures identified at the leaf level are supported by known processes that explain such crosstalks and moreover, provide evidence of numerous unknown interactions that still require exploration. To achieve this, transcriptomic analyses firstly at a global scale and secondly, with a focus on already identified ionomic genes (Whitt et al., 2020), could provide a way to explain ionomic modifications and the associated crosstalks between mineral nutrients. Finally, although we have explored the effects of most mineral deficiencies, other abiotic factors may differentially impact the ionome composition of plant tissues, including those generated by climatic changes. For example, the meta-analysis conducted by Loladze (2014) on FACE experiments showed that elevated CO_2_ impacts the plant ionome, mostly by shifting the mineral content downward. A lack of knowledge still exists about the effects of other abiotic stresses on leaf ionomic composition such as high temperatures with or without water deficit (Etienne et al., 2018).

## Data Availability Statement

The original contributions presented in the study are included in the article/Supplementary Material, further inquiries can be directed to the corresponding author/s.

## Author Contributions

AD’O, GC, AO, SD, PE, MA, and SP conceived and designed the experiments. AD’O, GC, and AL performed the greenhouse experiments and acquired the data. AD’O and GC analyzed the data. AD’O, GC, AO, and SD wrote the manuscript. PE, MA, AM, and SP revised the manuscript content. All authors contributed to the article and approved the submitted version.

## Conflict of Interest

The authors declare that the research was conducted in the absence of any commercial or financial relationships that could be construed as a potential conflict of interest.

## Supplementary Material

Supplementary Data 1 Compositions of the nutrient solutions derived from Hoagland nutrient solution and modified based on Maillard et al. (2016a) in order to grow hydroponic plants with an ionomic composition as close as possible to field observations (data not shown, see Courbet et al., 2021). These solutions were used to expose 22 days *Brassica napus* L. and *Triticum aestivum* to single nutrient deprivations of boron (B), chlorine (Cl), manganese (Mn), iron (Fe), nickel (Ni), copper (Cu), zinc (Zn), molybdenum (Mo), sodium (Na), silicon (Si), cobalt (Co), or selenium (Se).

Supplementary Data 2 Principal component analysis (PCA) score plots of the complete elemental content data. Projection of the data onto the subspace spanned by components 1 (PC 1) and and 2 (PC2), which are colored by species **(A)** or tissue **(B)** and each individual labeled with is treatment class (control or deprived plant). The contribution plots **(C,D)** depict the importance of each element in component 1 and 2, respectively, the bar length representing regression coefficients with either positive or negative signs. Variables are ranked by decreasing importance starting from the bottom.

Supplementary Data 3 Mineral nutrient concentrations of *B. napus* and *T. aestivum* control plants grown for 22 days under hydroponic conditions (D_22_). Tissues developed before or after D_0_ are indicated as “young” or “old” as follows: young leaf blades (YLBs), old leaf blades (OLBs), young petioles (YPs), and old petioles (OPs). Data are expressed in parts per million (ppm) and given as the mean with each replicate (*n* = 5) corresponding to a pool of two individual plants.

Supplementary Data 4 Classification parameters of each predicted class for rapeseed and wheat tissues. Results are presented as the mean (*n* = 10) from prediction method A considering the entire ionomic data (see section “Materials and Methods”).
