# Supplementary material for: Specificity and Plasticity of the Functional Ionome of Brassica napus and Triticum aestivum Exposed to Micronutrient or Beneficial Nutrient Deprivation and Predictive Sensitivity of the Ionomic Signatures
Source: Front Plant Sci. 2021 Feb 10;12:641678. doi: 10.3389/fpls.2021.641678 (PMC7902711; doi:10.3389/fpls.2021.641678)
Supplement: Supplementary Data 1 — Compositions of the nutrient solutions derived from Hoagland nutrient solution and modified based on Maillard et al. (2016a) in order to grow hydroponic plants with an ionomic composition as close as possible to field observations (data not shown, see Courbet et al., 2021). These solutions were used to expose 22 days Brassica napus L. and Triticum aestivum to single nutrient deprivations of boron (B), chlorine (Cl), manganese (Mn), iron (Fe), nickel (Ni), copper (Cu), zinc (Zn), molybdenum (Mo), sodium (Na), silicon (Si), cobalt (Co), or selenium (Se). [file Data_Sheet_1.pdf]

Supplemental data 1: Compositions of the nutrient solutions derived from Hoagland nutrient solution and modified based on Maillard *et al.* (2016a) in order to get hydroponic ionome as close as field observations (data not shown, see Courbet *et al.* (2021)). These solutions were used to expose 22 days *Brassica napus L*. and *Triticum aestivum* to unique nutrient deprivations in boron (B), chlorine (Cl), manganese (Mn), iron (Fe), nickel (Ni), copper (Cu), zinc (Zn), Molybdenum (Mo), sodium (Na), silicon (Si), cobalt (Co) or selenium (Se).

Salts	Salt concentrations (µM) of deprived solutions :												
	Control	-B	-Cl	-Mn	-Fe	-Ni	-Cu	-Zn	-Mo	-Na	-Si	-Co	-Se
KNO_3	1000	1000	1000	1000	1000	1000	1000	1000	1000	1000	1000	1000	1000
$Ca(NO_3)_2 + 4H_2O$	1250	1250	1250	1250	1250	1250	1250	1250	1250	1250	1250	1250	1250
KH_2PO_4	200	200	200	200	200	200	200	200	200	200	200	200	200
K_2HPO_4	0	0	0	0	0	0	0	0	0	0	0	0	0
$MgSO_4$	400	400	400	400	400	400	400	400	400	400	400	400	400
$MgCl_2$	0	0	0	0	0	0	0	0	0	0	0	0	0
NaFe-EDTA $3H_2O$	50	50	50	50	0	50	50	50	50	0	50	50	50
NaFe-EDDHA	50	50	50	50	0	50	50	50	50	0	50	50	50
Fe-Citrate	0	0	0	0	0	0	0	0	0	100	0	0	0
H_3BO_3	10	0	10	10	10	10	10	10	10	10	10	10	10
$MnSO_4$	3	3	3	0	3	3	3	3	3	3	3	3	3
$MnCl_2$	0	0	0	0	0	0	0	0	0	0	0	0	0
$ZnSO_4$	3	3	3	3	3	3	3	0	3	3	3	3	3
$CuSO_4$	0.7	0.7	0.7	0.7	0.7	0.7	0	0.7	0.7	0.7	0.7	0.7	0.7
$(NH_4)_6Mo_7O_{24}$	0.008	0.008	0.008	0.008	0.008	0.008	0.008	0.008	0	0.008	0.008	0.008	0.008
$CoCl_2$	0.1	0.1	0.1	0.1	0.1	0.1	0.1	0.1	0.1	0.1	0.1	0	0.1
$NiCl_2$	0.15	0.15	0.15	0.15	0.15	0	0.15	0.15	0.15	0.15	0.15	0.15	0.15
$Si(OH)_4$	900	900	900	900	850	900	900	900	900	1100	0	900	900
$CaCl_2 + 2H_2O$	500	500	0	500	500	500	500	500	500	500	500	500	500
KCl	100	100	0	100	100	100	100	100	100	100	100	100	100
Na_2SeO_4	0.01	0.01	0.01	0.01	0.01	0.01	0.01	0.01	0.01	0.01	0.01	0.01	0
$CaCO_3$	360	360	360	360	360	360	360	360	360	360	360	360	360
K_2SO_4	100	100	100	100	100	100	100	100	100	100	100	100	100
Na_2SiO_3	200	200	200	200	250	200	200	200	200	0	0	200	200
HCl	600	600	0	600	660	600	600	600	600	0	300	600	600
H3PO4	0	0	300	0	0	0	0	0	0	0	0	0	0

Nutrients	Elemental concentrations (µM) of deprived solutions :												
	Control	-B	-Cl	-Mn	-Fe	-Ni	-Cu	-Zn	-Mo	-Na	-Si	-Co	-Se
N	3500	3500	3500	3500	3500	3500	3500	3500	3500	3500	3500	3500	3500
Mg	400	400	400	400	400	400	400	400	400	400	400	400	400
P	200	200	500	200	200	200	200	200	200	200	200	200	200
S	507	507	507	504	507	507	506	504	507	507	507	507	507
K	1500	1500	1400	1500	1500	1500	1500	1500	1500	1500	1500	1500	1500
Ca	2110	2110	1610	2110	2110	2110	2110	2110	2110	2110	2110	2110	2110
B	10	0	10	10	10	10	10	10	10	10	10	10	10
Cl	2001	1701	1	1701	1761	1700	1701	1701	1701	1101	1401	1700	1701
Mn	3	3	3	0	3	3	3	3	3	3	3	3	3
Fe	100	100	100	100	0	100	100	100	100	100	100	100	100
Ni	0.15	0.15	0.15	0.15	0.15	0	0.15	0.15	0.15	0.15	0.15	0.15	0.15
Cu	0.7	0.7	0.7	0.7	0.7	0.7	0	0.7	0.7	0.7	0.7	0.7	0.7
Zn	3	3	3	3	3	3	3	0	3	3	3	3	3
Mo	0.056	0.056	0.056	0.056	0.056	0.056	0.056	0.056	0.0	0.056	0.056	0.056	0.056
Na	500	500	500	500	500	500	500	500	500	0	100	500	500
Al													
Si	1100	1100	1100	1100	1100	1100	1100	1100	1100	1100	0	1100	1100
V													
Co	0.1	0.1	0.1	0.1	0.1	0.1	0.1	0.1	0.1	0.1	0.1	0	0.1
Se	0.01	0.01	0.01	0.01	0.01	0.01	0.01	0.01	0.01	0.01	0.01	0.01	0
