# Supplementary material for: Specificity and Plasticity of the Functional Ionome of Brassica napus and Triticum aestivum Exposed to Micronutrient or Beneficial Nutrient Deprivation and Predictive Sensitivity of the Ionomic Signatures
Source: Front Plant Sci. 2021 Feb 10;12:641678. doi: 10.3389/fpls.2021.641678 (PMC7902711; doi:10.3389/fpls.2021.641678)

Supplemental data 2: Principal component analysis (PCA) score plots of the complete elemental content data. Projection of the data onto the subspace spanned by components 1 (PC 1) and 2 (PC2), which are colored by species **(A)** or tissue **(B)** and each individual labeled with is treatment class (control or deprived plant). The contribution plots **(C,D)** depict the importance of each element in component 1 and 2, respectively, the bar length representing regression coefficients with either positive or negative signs. Variables are ranked by decreasing importance starting from the bottom.

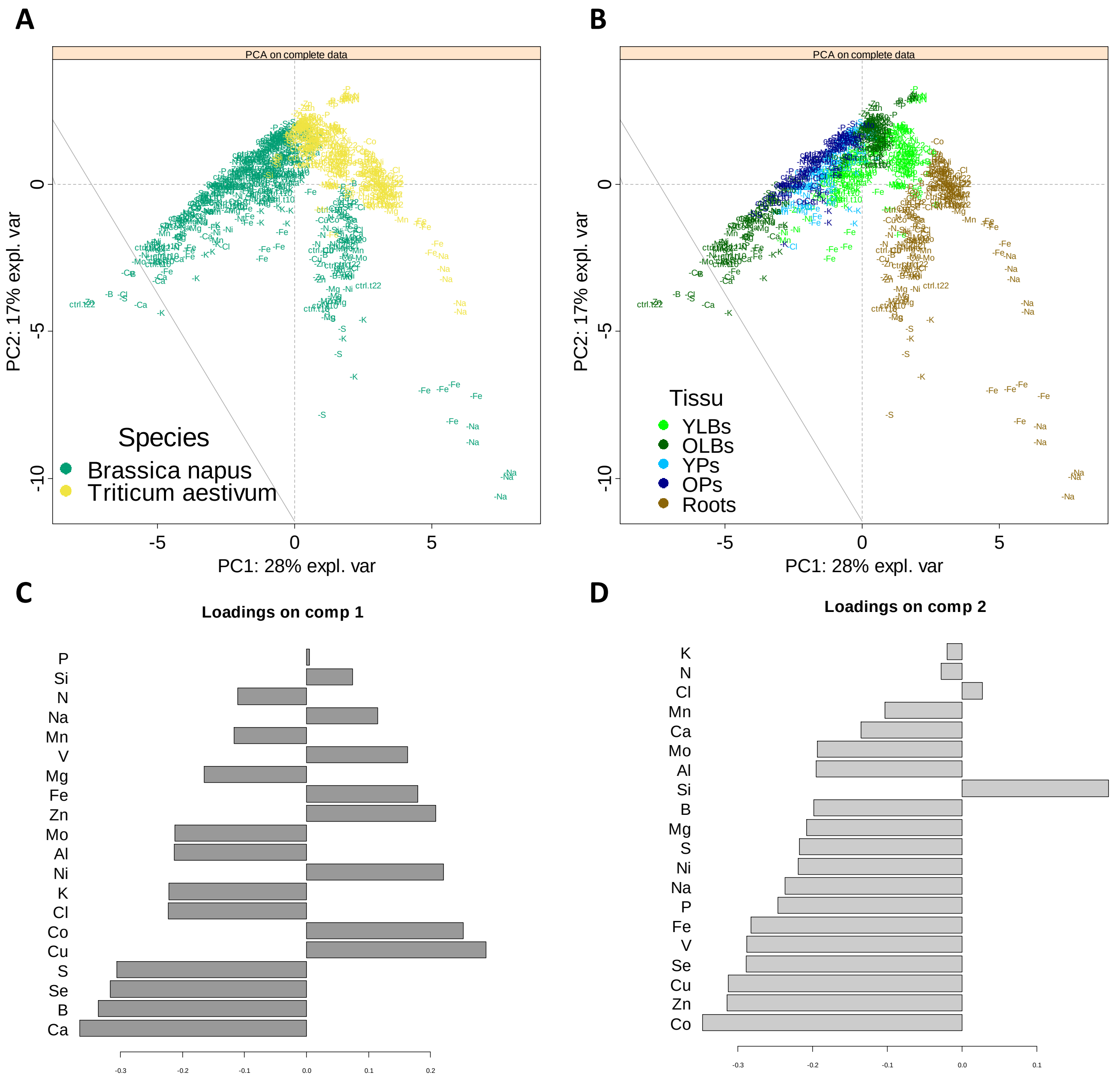

Supplement: Supplementary Data 2 — Principal component analysis (PCA) score plots of the complete elemental content data. Projection of the data onto the subspace spanned by components 1 (PC 1) and and 2 (PC2), which are colored by species (A) or tissue (B) and each individual labeled with is treatment class (control or deprived plant). The contribution plots (C,D) depict the importance of each element in component 1 and 2, respectively, the bar length representing regression coefficients with either positive or negative signs. Variables are ranked by decreasing importance starting from the bottom. [file Data_Sheet_2.pdf]
