# Supplementary material for: Specificity and Plasticity of the Functional Ionome of Brassica napus and Triticum aestivum Exposed to Micronutrient or Beneficial Nutrient Deprivation and Predictive Sensitivity of the Ionomic Signatures
Source: Front Plant Sci. 2021 Feb 10;12:641678. doi: 10.3389/fpls.2021.641678 (PMC7902711; doi:10.3389/fpls.2021.641678)
Supplement: Supplementary Data 3 — Mineral nutrient concentrations of B. napus and T. aestivum control plants grown for 22 days under hydroponic conditions (D22). Tissues developed before or after D0 are indicated as “young” or “old” as follows: young leaf blades (YLBs), old leaf blades (OLBs), young petioles (YPs), and old petioles (OPs). Data are expressed in parts per million (ppm) and given as the mean with each replicate (n = 5) corresponding to a pool of two individual plants. [file Data_Sheet_3.pdf]

Supplemental data 3: Mineral nutrient concentrations of *B. napus* and *T. aestivum* control plants grown 22 days under hydroponic conditions (D_{22}). Tissues developed before or after D_0 are indicated as "young" or "old" as follows: young leaf blades (YLBs), old leaf blades (OLBs), young petioles (YPs) and old petioles (OPs). Data are expressed in parts per million (ppm) and given as the mean with each replicate (n=5) corresponding to a pool of two individual plants.

		Brassica napus					***Triticum aestivum***		
		**YLB**	**OLB**	**YP**	**OP**	**roots**	**YLB**	**OLB**	**roots**
Macronutrients	**N**	51 222	31 856	27 041	21 206	26 307	30 540	21 141	18 909
	**Mg**	2 545	5 448	3 767	3 818	3 240	1 655	3 701	4 355
	**P**	6 650	3 427	6 132	3 315	5 819	4 583	1 630	3 400
	**S**	11 143	22 794	7 486	5 800	7 568	4 079	6 501	2 843
	**K**	42 453	51 139	85 501	69 525	45 327	45 098	35 874	19 969
	**Ca**	17 344	63 343	28 246	37 260	7 169	4 441	15 871	2 649
Micronutrients	**B**	59	101	45	33	24	8	10	5
	**Cl**	9 697	19 794	27 702	29 566	12 606	12 436	13 142	10 029
	**Mn**	93	244	61	62	81	65	280	87
	**Fe**	115	114	59	44	841	208	138	639
	**Ni**	0.3	0.7	0.6	0.5	5.8	9.0	0.5	3.9
	**Cu**	6	4	4	2	33	9	6	47
	**Zn**	52	31	33	28	72	59	66	62
	**Mo**	3.9	6.5	3.0	4.1	4.3	4.0	4.6	0.7
Beneficial nutrients	**Na**	714	1 250	2 837	3 743	9 163	530	569	8 922
	**Al**	469	667	682	705	445	346	358	301
	**Si**	882	1 309	685	708	2 721	9 786	46 200	3 368
	**V**	0.04	0.07	0.06	0.02	0.34	0.13	0.04	0.12
	**Co**	0.12	0.15	0.09	0.08	6.34	0.29	0.11	4.89
	**Se**	0.66	1.40	0.74	0.62	0.58	0.46	0.41	0.28
