# Supplementary material for: Specificity and Plasticity of the Functional Ionome of Brassica napus and Triticum aestivum Exposed to Micronutrient or Beneficial Nutrient Deprivation and Predictive Sensitivity of the Ionomic Signatures
Source: Front Plant Sci. 2021 Feb 10;12:641678. doi: 10.3389/fpls.2021.641678 (PMC7902711; doi:10.3389/fpls.2021.641678)
Supplement: Supplementary Data 4 — Classification parameters of each predicted class for rapeseed and wheat tissues. Results are presented as the mean (n = 10) from prediction method A considering the entire ionomic data (see section “Materials and Methods”). [file Data_Sheet_4.pdf]

Supplemental data SD 4: Classification parameters of each predicted class for rapeseed and wheat tissues. Results are presented as the mean (n= 10) from prediction method A considering the entire ionomic data (see section “Materials and Methods”).

					Nutrient deficiency																	
			**control D10**	**control D22**	**-N**	**-Mg**	**-P**	**-S**	**-K**	**-Ca**	**-B**	**-Cl**	**-Mn**	**-Fe**	**-Ni**	**-Cu**	**-Zn**	**-Mo**	**-Na**	**-Si**	**-Co**	**-Se**
Brassica napus	YLBs	Sensitivity	0.9	0.9	1	1	1	1	1	1	1	1	1	1	0.4	0.9	1	1	1	1	0.7	1
		Specificity	1	0.98	1	1	0.99	1	1	1	1	0.98	0.99	1	1	1	1	1	1	0.99	0.99	1
		Balanced Accuracy	0.95	0.94	1	1	0.997	1	1	1	1	0.992	0.997	1	0.70	0.95	1	1	1	0.99	0.85	1
	OLBs	Sensitivity	1	0.5	0.9	0.8	1	1	1	1	1	1	1	1	0.7	0.8	0.9	1	0.9	0.9	1	1
		Specificity	1	0.98	1	1	1	0.98	1	1	1	1	1	0.99	0.99	0.98	0.99	1	1	0.99	0.99	0.99
		Balanced Accuracy	1	0.74	0.95	0.9	1	0.99	1	1	1	1	1	1	0.85	0.89	0.95	1	0.95	0.95	0.99	1
	Roots	Sensitivity	0.5	0.3	0.7	0.7	0.7	0.7	0.7	0.7	0.5	0.7	0.7	0.7	0.7	0.7	0.7	0.7	0.7	0.7	0.8	0.5
		Specificity	0.99	0.99	0.99	0.99	0.99	0.99	0.99	0.99	0.99	0.99	0.97	0.99	0.99	0.99	0.99	0.99	0.99	0.98	0.97	0.99
		Balanced Accuracy	0.75	0.65	0.84	0.84	0.84	0.84	0.84	0.84	0.74	0.84	0.84	0.84	0.84	0.84	0.84	0.84	0.84	0.84	0.89	0.74
	YPs	Sensitivity	1	0.7	1	1	1	1	1	1	1	0.7	1	1	0.7	0.9	1	1	1	0.9	1	1
		Specificity	1	0.99	1	1	1	1	1	0.98	1	1	0.98	1	0.99	1	0.99	1	1	1	1	0.99
		Balanced Accuracy	1	0.84	1	1	1	1	1	0.99	1	0.85	0.99	1	0.85	0.95	1	1	1	0.95	1	1
	Ops	Sensitivity	1	0.2	1	1	1	1	1	1	1	1	1	1	0.8	0.6	1	1	1	0.7	1	0.9
		Specificity	1	0.98	0.99	1	1	1	1	1	1	1	1	1	1	0.96	1	1	1	0.99	0.99	0.98
		Balanced Accuracy	1	0.59	0.99	1	1	1	1	1	1	1	1	1	0.9	0.78	1	1	1	0.85	1	0.94
Triticum aestivum	YLBs	Sensitivity	1	0.8	1	1	1	1	1	1	0.9	0.9	1	1	0.4	1	1	0.9	1	1	0.9	1
		Specificity	1	0.99	0.99	1	1	1	1	1	1	1	1	1	0.99	1	1	0.99	1	1	0.99	0.98
		Balanced Accuracy	1	0.89	0.997	1	1	1	1	1	0.95	0.95	1	1	0.70	1	1	0.94	1	1	0.94	0.99
	Ops	Sensitivity	1	1	1	1	1	1	1	1	1	1	1	1	0.4	0.9	1	1	0.7	1	0.1	0.6
		Specificity	1	0.98	1	1	1	1	1	1	0.96	1	1	0.99	1	0.97	0.99	0.99	1	1	0.99	0.99
		Balanced Accuracy	1	0.99	1	1	1	1	1	1	0.98	1	1	1	0.7	0.93	1	0.99	0.85	1	0.54	0.8
	Roots	Sensitivity	1	0.9	1	1	1	1	1	1	1	0.9	1	1	0.8	1	1	1	1	0.9	1	0.7
		Specificity	1	1	1	1	1	1	0.99	1	0.99	1	1	1	0.99	0.99	1	0.98	1	1	1	0.99
		Balanced Accuracy	1	0.95	1	1	1	1	1	1	1	0.95	1	1	0.9	1	1	0.99	1	0.95	1	0.85
